# Characterization of bacterioplankton communities from a hatchery recirculating aquaculture system (RAS) for juvenile sole (*Solea senegalensis*) production

**DOI:** 10.1371/journal.pone.0211209

**Published:** 2019-01-25

**Authors:** Letícia N. Duarte, Francisco J. R. C. Coelho, Vanessa Oliveira, Daniel F. R. Cleary, Patrícia Martins, Newton C. M. Gomes

**Affiliations:** Department of Biology & CESAM, University of Aveiro, Aveiro, Portugal; Universitat Politècnica de València, SPAIN

## Abstract

There is a growing consensus that future technological developments of aquaculture systems should account for the structure and function of microbial communities in the whole system and not only in fish guts. In this study, we aimed to investigate the composition of bacterioplankton communities of a hatchery recirculating aquaculture system (RAS) used for the production of Senegalese sole (*Solea senegalensis*) juveniles. To this end, we used a 16S rRNA gene based denaturing gradient gel electrophoresis (DGGE) and pyrosequencing analyses to characterize the bacterioplankton communities of the RAS and its water supply. Overall, the most abundant orders were Alteromonadales, Rhodobacterales, Oceanospirillales, Vibrionales, Flavobacteriales, Lactobacillales, Thiotrichales, Burkholderiales and Bdellovibrionales. Although we found a clear distinction between the RAS and the water supply bacterioplankton communities, most of the abundant OTUs (≥50 sequences) in the hatchery RAS were also present in the water supply. These included OTUs related to *Pseudoalteromonas* genus and the Roseobacter clade, which are known to comprise bacterial members with activity against Vibrio fish pathogens. Overall, in contrast to previous findings for sole grow-out RAS, our results suggest that the water supply may influence the bacterioplankton community structure of sole hatchery RAS. Further studies are needed to investigate the effect of aquaculture practices on RAS bacterioplankton communities and identification of the key drivers of their structure and diversity.

## Introduction

The world population is expected to reach approximately 9.7 billion in 2050 [1]. As population increases, so will the demand for food, which will have to increase by 70% over the period from 2005–2050 [2]. The increase in demand will require substantial technological advances in food production. At present, aquaculture is undergoing rapid technological development and is emerging as a major food production sector. The demand for higher sustainability, reduced production costs and food safety has continuously driven the development of new and innovative aquaculture systems. Technologies such as recirculating aquaculture systems (RAS) with shallow raceway systems (SRS) allow more controlled and cost-effective production conditions, while having a reduced environmental impact. RAS is an advanced approach that reuses water in the production system with mechanical and biological filters [3]. SRS contribute for an optimized hydrodynamic performance over common raceways, allowing a lower water level and plug-flow pattern that enables high fish stocking densities, improving overall productivity [4]. RAS technology with shallow raceways continuously processes and recycles water, reducing water pump requirements while maintaining optimal environmental conditions for fish production [4]. However, the use of high fish densities during production may result in more rapid and severe disease outbreaks [5]. In fact, currently, there is a growing understanding that improvements in the prevention and management of disease outbreaks requires a deeper knowledge of the ecology of microbial communities in aquaculture systems. Outbreaks of parasitic, bacterial, and fungal diseases are among the most important limiting factors for the success of aquaculture production, leading to high mortality rates and important economic losses [6]. For example, the production of Senegalese sole (*Solea senegalensis*), a species of considerable commercial value, is strongly limited by its sensitivity to infectious diseases such as pasteurellosis (caused by *Photobacterium damselae* subsp *piscicida*), vibriosis (caused by various species of the genus *Vibrio*, especially *Vibrio anguillarum*) and flexibacteriose (caused by *Tenacibaculum maritimum*) [7]. However, despite the deleterious effects of fish pathogens, the aquaculture water microbiome is essential for maintaining water quality (nutrient recycling) and fish health during intensive fish production [8, 9]. For example, nitrogen and phosphorus are recycled through the activity of heterotrophic decomposers [10]. The presence of beneficial microbes was also shown to reduce colony-forming units (CFU) of pathogenic bacterial species [11]. Naturally occurring or introduced beneficial bacteria (probiotics) may contribute to improve water quality, inhibit the development of fish pathogens, improve the fish immune system and promote the balance of the fish bacterial flora [8, 12, 13].

In previous studies, we showed that *S*. *senegalensis* appears to influence the bacterial communities in a grow-out RAS and that, despite the presence of several potential fish pathogens, no diseased fish were observed during the study period. Our findings indicated that the water in grow-out RAS was dominated by naturally occurring beneficial microbes (antagonistic populations), which may have played an important role in suppressing the development of putative pathogens [12, 14]. However, we could not determine if such a trend would also be detectable in RAS systems used for production of juvenile specimens (hatchery), which are managed under different conditions and stocked from hatching until juvenile stage. Here, we aimed to investigate bacterioplankton community composition and diversity in the water of a commercial hatchery operating a RAS for the production of sole (*S*. *senegalensis*) juveniles and compare results with those previously recorded for sole grow-out RAS [12]. We also evaluated our results in light of the putative function of bacterioplankton populations in the hatchery RAS.

## Material and methods

### Study site and experimental design

Fieldwork was conducted in October 2013 in a RAS at a hatchery employing SRS for juvenile Senegalese sole with a capacity to produce more than 1 million juveniles per year that are stocked from hatching until they reach approximately 40 g. The fish hatchery employed water recirculation at a renewal rate of <5% of total system volume per day. Briefly, the water supply reservoir (Sup) is filled with seawater pumped through an inlet pipe from the ocean and is ozonized in a tank connected to a protein skimmer (Ozo) before entering the pre-production reservoir (Pre) (hatchery containing juvenile sole weighing approximately 4 g and densities with about 3.7 kg/m^2^). Water from Pre is recycled by passing through a sedimentation tank (Sed) where mechanical filtration is also carried out. After mechanical filtration, water flows to a biofilter tank (Bio) for biological filtration and is subsequently pumped back to Ozo where it reenters the system. Water samples for bacterial community analysis and chemical characterization were collected in triplicate from all 5 different compartments (Sup, Ozo, Pre, Sed and Bio). Samples were obtained in the aquaculture company with permission from legal representatives.

### Water chemistry analysis and bacterial communities

#### Chemical analysis

Ammonium (NH_4_^+^), nitrites (NO_2_^-^) and nitrates (NO_3_^-^) were determined for each water sample collected following the NP 730, EPA 300.1 and NP EN 26777 methods, respectively. Bromide (Br-) was determined according to EPA Method 300.1. Total organic carbon analysis (TOC) in the water was performed according to the European Norm 1484. Conventional physicochemical parameters, namely, temperature, pH, dissolved oxygen (DO) and salinity were also measured.

#### DNA analysis

Water samples were transported to the laboratory and immediately processed for DNA extraction. Briefly, 250 ml of water were filtered through 0.2μm pore size polycarbonate membranes (Poretics, Livermore, CA, USA) and total DNA was extracted from each filter using the E.Z.N.A. Soil DNA Extraction kit (Omega Bio-Tek, USA) according to the manufacturer’s instructions. Bacterial community composition was compared among samples using DGGE fingerprinting in combination with a more-in-depth barcoded pyrosequencing analysis of composite samples [15]. Amplified 16S rRNA gene fragments suitable for bacterial DGGE fingerprints of total microbial community DNA samples were obtained using a nested approach following Gomes *et al*. (2008) [16]. In the first PCR, amplicons of the bacterial 16S rRNA gene were obtained using bacteria specific primers 27F and 1494R (21 PCR cycles) [17]. For DGGE analyses, the second PCR (21 PCR cycles) used the primers 968GC - 1378R [18], with a GC clamp attached to the 5’ end to prevent complete melting of double-stranded DNA during DGGE. DGGE was performed on a DCode Universal Mutation Detection System (Bio-Rad, Hercules, CA, USA), in 1x Trisacetate-EDTA (TAE) with a denaturing gradient ranging from 40% to 58% (100% denaturant contains 7 M urea and 40% formamide) and performed at 58°C at 160 V during 16 hours onto 8% (w/v) polyacrylamide gels. DGGE gels were silver stained as described by Byun *et al*. (2009) [19], except for the stop solution that was replaced by a NaHCO_3_ 9% solution. The image was acquired using an Epson perfection V700 Photo Scanner. Digitalized DGGE gels were analysed with the software Bionumerics (Applied Maths). Briefly, both band position and intensity were processed in a spreadsheet. The data matrix of relative abundance (band positions and their corresponding intensities) per sample was log_10_ (*x* +1) transformed, and a distance matrix was constructed using the Bray-Curtis similarity coefficient with the vegdist() function in the vegan package [20] in R (version 3.1.1; http://www.r-project.org/). Variation in bacterial composition among compartments was visually assessed with principal coordinates analysis (PCO) using the cmdscale() function in R using the Bray-Curtis distance matrix as input.

For compositional analysis, DNA from the three replicates of each compartment was pooled to obtain one DNA library per compartment. The V3-V4 regions of the 16S rRNA gene were amplified using barcoded fusion primers V3 Forward (5´ -ACTCCTACGGGAGGCAG-3’) and V4 Reverse (5´ -TACNVRRGTHTCTAATYC-3’) [21]. The amplified fragments were purified (Agencourt Ampure beads, Agencourt Bioscience Corporation, MA, USA) and then sequenced using a Roche 454 FLX Titanium pyrosequencer (Brandford, CT, USA) following manufacturer’s guidelines. Sequencing was performed at MR DNA (www.mrdnalab.com, Shallowater, TX, USA).

The barcoded pyrosequencing libraries were processed using the QIIME (Quantitative Insights Into Microbial Ecology; [22]) software package (http://qiime.org; accessed 15/03/2014) according to published recommendations [23] and following previously described methods [24, 25], with the exception of the OTU picking step (97% threshold), where the UPARSE [26] clustering method and chimera check were used, and the most recent Greengenes database (ftp://greengenes.microbio.me/greengenes_release/gg_13_5/gg_13_8_otus.tar.gz) for OTU picking and taxonomic assignment. Full details about the UPARSE steps can be found in Cleary *et al*. (2015) [24]. Finally, the make_otu_table.py script was used to produce an OTU by sample table containing the abundance and taxonomic assignment of all OTUs. After removal of non-bacteria, chloroplasts and mitochondria sequences, this table was uploaded to R software (version 3.1.1; http://www.r-project.org/) for statistical computing and graphics.

Rarefaction curves were made for each sampling compartment using a self-written function in R [27]. Variation in OTU composition was visualized using principal coordinates analysis (PCO) with the cmdscale() function in R. Variation in the relative abundance of the most abundant bacterial taxa was assessed using barplot graphs. In addition to this, OTUs taxonomically classified into genera which often comprise potential fish pathogens were selected and representative sequences compared with those available in GenBank. We used BLAST search (GenBank Nucleotide Databases Searched http://www.ncbi.nlm.nih.gov/) to obtain the closest relatives of selected OTUs (pathogens and abundant taxa, i.e., number of sequences ≥ 50). Sequences were, furthermore, aligned using ClustalW and a phylogenetic tree was constructed in Mega7 (http://www.megasoftware.net/) using the Maximum Composite Likelihood method with a gamma distribution (five categories) and 1000 bootstraps to compute evolutionary distances. The iTOL v3 (http://itol.embl.de/) server was used to annotate the phylogenetic tree [28]. DNA sequences generated in this study have been submitted to the NCBI SRA (Accession number SRP095444).

## Results and discussion

The physicochemical characteristics of the water in each compartment are summarized in Table 1. The most notable differences were between Sup and the hatchery RAS compartments. There was a slight increase in pH and fairly low levels of nutrients in the Sup compartment when compared to RAS compartments (Table 1).

**Table 1:** Physico-chemical parameters in the pre-production RAS for each sampling point.

	Temperature °C	pH	DO mg/L	Salinity	Ammonium mgNH_4_^+^/L	Nitrite mgNO_2_^-^/L	Nitrate mgNO_3_^-^/L	Bromide mgBr/L	TOC mg/L
**Sup**	19.1	7.95 ± 0.03	7.82	35	0.57 ± 0.51	< 1.00 *	0.97 ± 0.87	0.00	1.30 ± 0.10
**Pre**	20.2	7.18 ± 0.00	16.86	35	0.90 ± 0.00	4.40 ± 0.00	19.40 ± 0.69	0.06	4.67 ± 1.15
**Sed**	20.3	7.23 ± 0.02	9.77	35	0.60 ± 0.53	4.50 ± 0.00	19.20 ± 0.36	0.07	4.67 ± 1.15
**Bio**	20.3	7.30 ± 0.03	7.90	35	0.73 ± 0.06	4.63 ± 0.06	19.93 ± 0.40	0.07	4.00 ± 0.00
**Ozo**	20.3	7.33 ± 0.00	20.00	35	0.67 ± 0.06	4.43 ± 0.11	20.03 ± 0.32	0.09	4.00 ± 0.00

Sup—water supply, Ozo—ozonation tank, Bio—biofilter tank, Pre—pre-production (hatchery) tank and Sed—sedimentation tank.

* concentration below the limit of quantification

DO concentration ranged from 7.82 mg/L in Sup to 20 mg/L in Ozo. Ammonia concentration was lowest in Sup (0.57±0.51 mg/L) and highest in Pre (0.90mg/L). Nitrite and nitrate concentrations were lower in Sup (<1 and 0.97±0.87 mg/L, respectively) when compared to RAS compartments, (average of 4.49±0.10 mg NO_2_/L and 19.64±0.40 mgNO_3_/L). We did not detect bromide in the Sup compartment and its concentration was stable in the hatchery system (average of 0.07±0.01 mg/L). TOC concentration was lower in Sup (1.3±0.10 mg/L) than in the other compartments (average of 4.33±0.78 mg/L). Overall, the concentration of nutrients in the sole hatchery was much lower than in the sole grow-out RAS characterized in our previous study [12]. Such a difference in nutrient levels may be expected, as juvenile fish are grown to adulthood in the grow-out RAS and, therefore, the system is exposed to higher loads of non-eaten feed and fish excretion.

**Fig 1:**
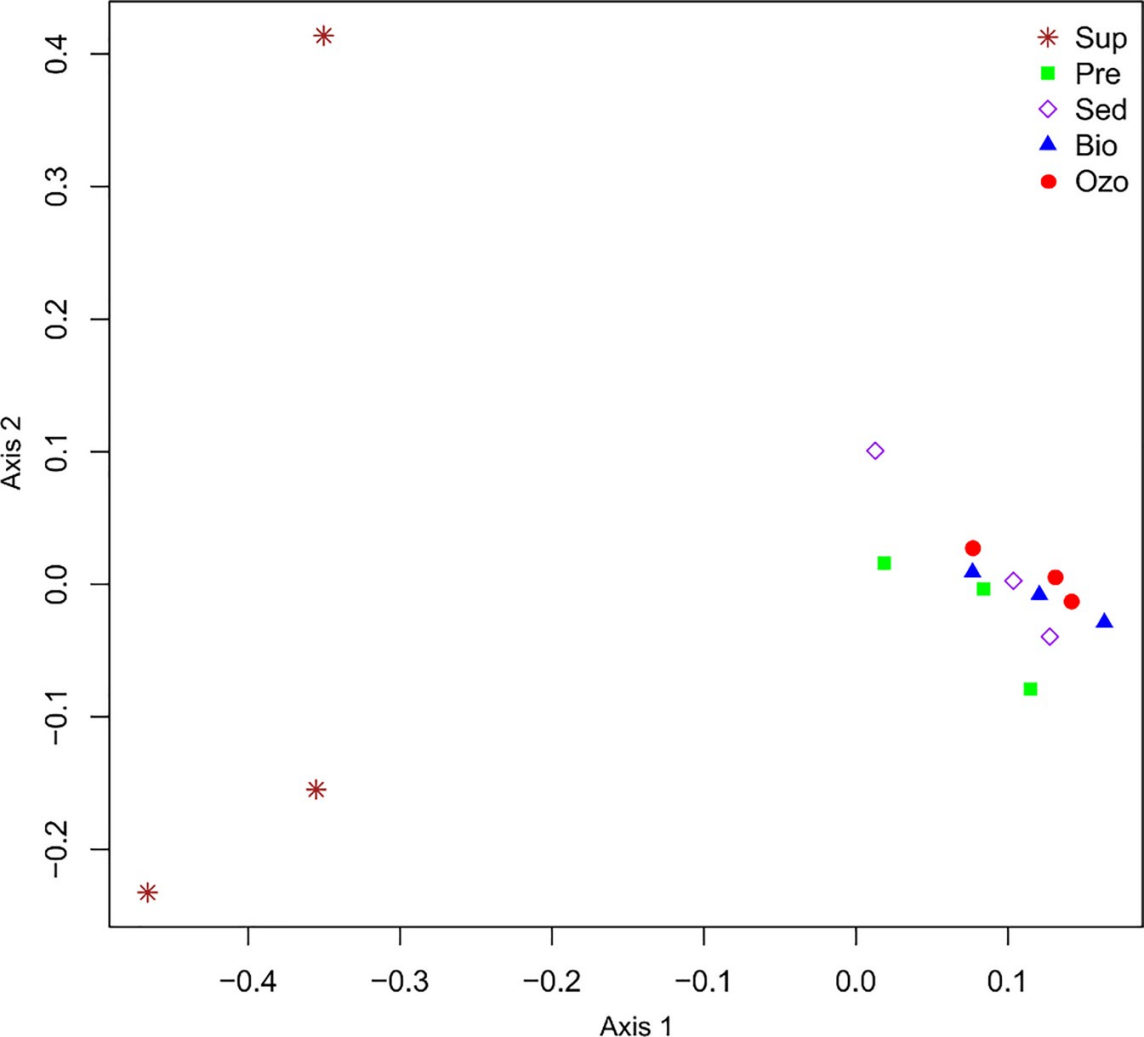
Principal Coordinates Analysis (PCO) of bacterial DGGE profiles. The first two explanatory axes are shown. Sup—water supply, Ozo—ozonation tank, Bio—biofilter tank, Pre—pre-production (hatchery) tank and Sed—sedimentation tank.

**Fig 2:**
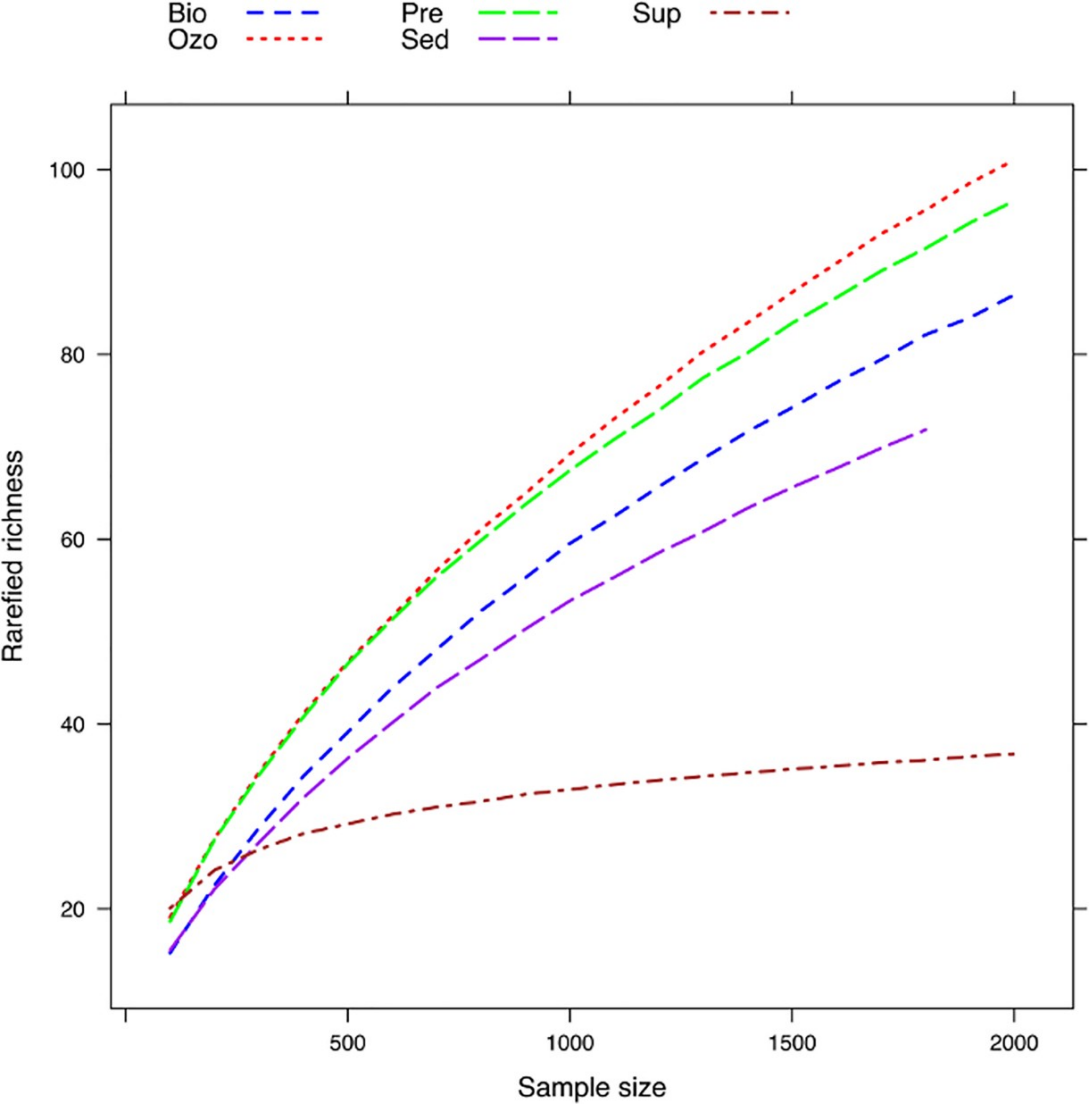
Rarefied OTU richness in all sampling compartments. Sup—water supply, Ozo—ozonation tank, Pre—pre-production tank, Sed—sedimentation tank and Bio—biofilter tank.

DGGE analysis of bacterioplankton communities showed that, despite the young age of fishes and their relatively short period in the tanks (45 days), there was a significant separation between water supply and RAS compartments (adonis; F_4,14_ = 2.831, R^2^ = 0.531, P = 0.003) (Fig 1). The communities of RAS compartments defined by DGGE also tended to cluster together (S1 Fig). The in-depth pyrosequencing analysis of these communities yielded a total of 14451 sequences that varied between 1858 in Sed to 4336 in the Ozo compartment. To examine changes in bacterial richness, rarefaction curves were generated for all compartments (Fig 2). Controlling for sampling size (*n* = 1700), OTU richness in the Sup compartment was 35.79±1.02. In the aquaculture tanks, richness was lowest in Sed (69.84±1.74) and highest in Ozo (92.88±4.82). The high diversity detected in Ozo may be due to an important fraction of dead microorganisms that accumulate in this compartment naturally derived from water supply and fish and feed waste from Pre tank and from the bacteria that proliferate in the biofilter. The introduction of ozone into a recirculation system is used to inactivate fish pathogens, remove accumulated organic residues and nitrite [29]. Ozonation has been showed to kill or inactivate fish pathogens and total heterotrophic bacterial loading [30, 31]. The effectiveness of ozone treatment, however, depends on ozone concentration, duration of ozone exposure, pathogen loads and levels of organic matter [29]; microorganisms able to persist following ozone treatment may again enter and grow in the system. DNA based analyses performed in this study, however, cannot provide any information on cell viability. Therefore, we cannot provide any information about the efficiency of ozone treatment on bacterial cell viability.

**Fig 3:**
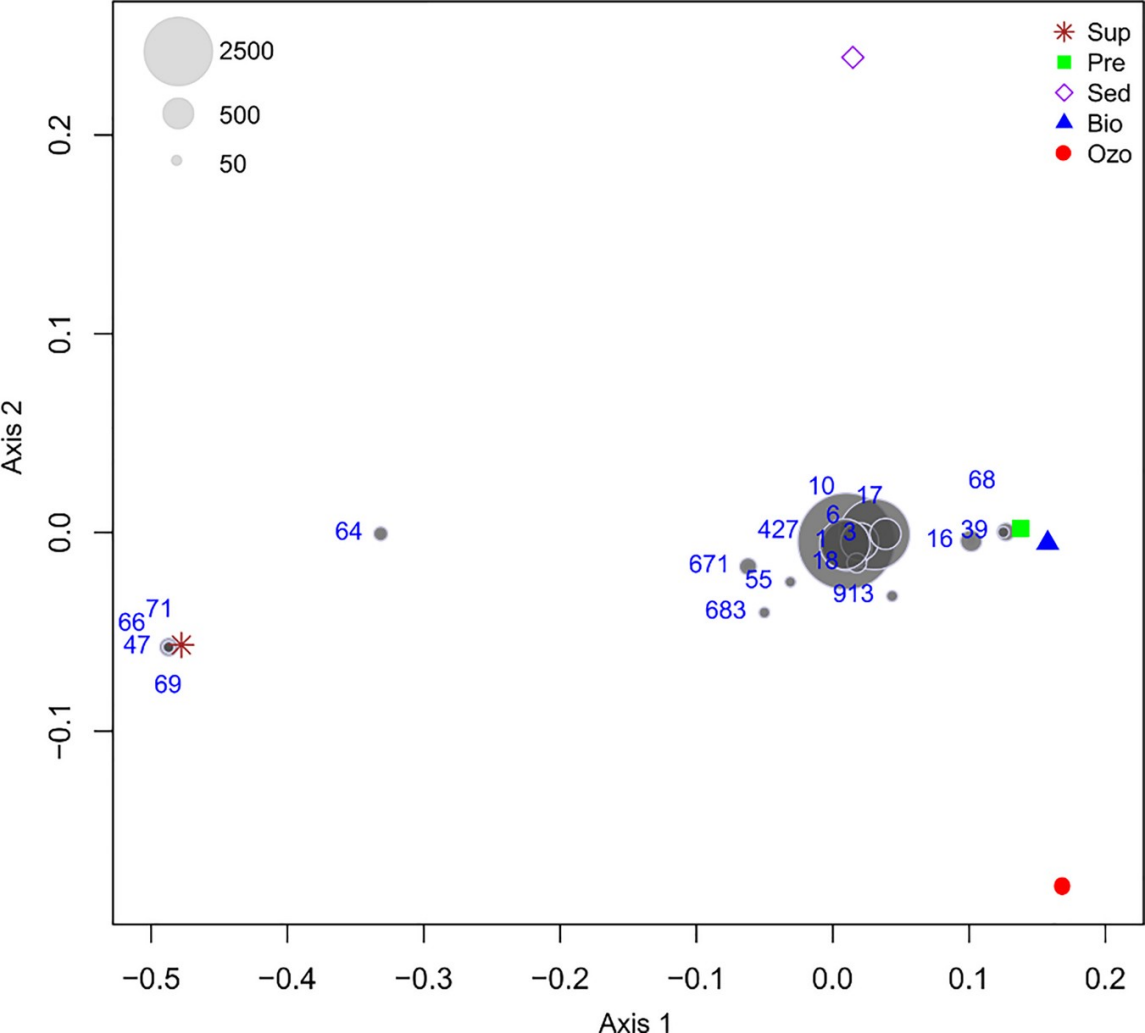
Ordination showing the first two axes of the Principal Coordinates Analysis (PCO) of bacterial OTU composition. The light gray symbols represent most abundant OTUs (≥50 sequences) with symbol size representing their abundance in the entire data set. Sup—water supply, Ozo—ozonation tank, Pre—pre-production tank, Sed—sedimentation tank and Bio—biofilter tank.

In line with the DGGE and richness analysis, the PCO ordination of OTU composition showed marked differences between water supply and RAS compartments (Fig 3). Along the first PCO axis, the Sup compartment separated from RAS compartments with a range of dominant OTUs shared by all compartments. These results indicate that, despite the fact that the bacterioplankton communities in the water supply were clearly distinct from RAS tanks, several dominant bacterial communities in the hatchery tanks were originally introduced in the system through the water supply. This finding is in contrast with the results obtained for sole grow out RAS [12], where only few bacterial OTUs were found to be dominant in the water supply and fish tanks. Probably, due to the early life stage development of the fish in this study, gut microbes released to the environment via feces may have had lower influence on hatchery water bacterioplankton than in grow out RAS. However, no fish gut samples were taken during this experiment, which hamper our ability to evaluate the contribution of fish microbiome to the hatchery bacterioplankton composition (and vice versa). Nevertheless, in line with this hypothesis, Giatsis *et al*. (2015) [32] showed that variations in gut bacterial community composition during Nile tilapia larvae (*Oreochromis niloticus*, Linnaeus) development were highly correlated with shifts in the bacterioplankton communities. Providing evidences that intestinal microbiota of the fish juveniles may share more similarities with their respective water bacterial communities.

**Fig 4:**
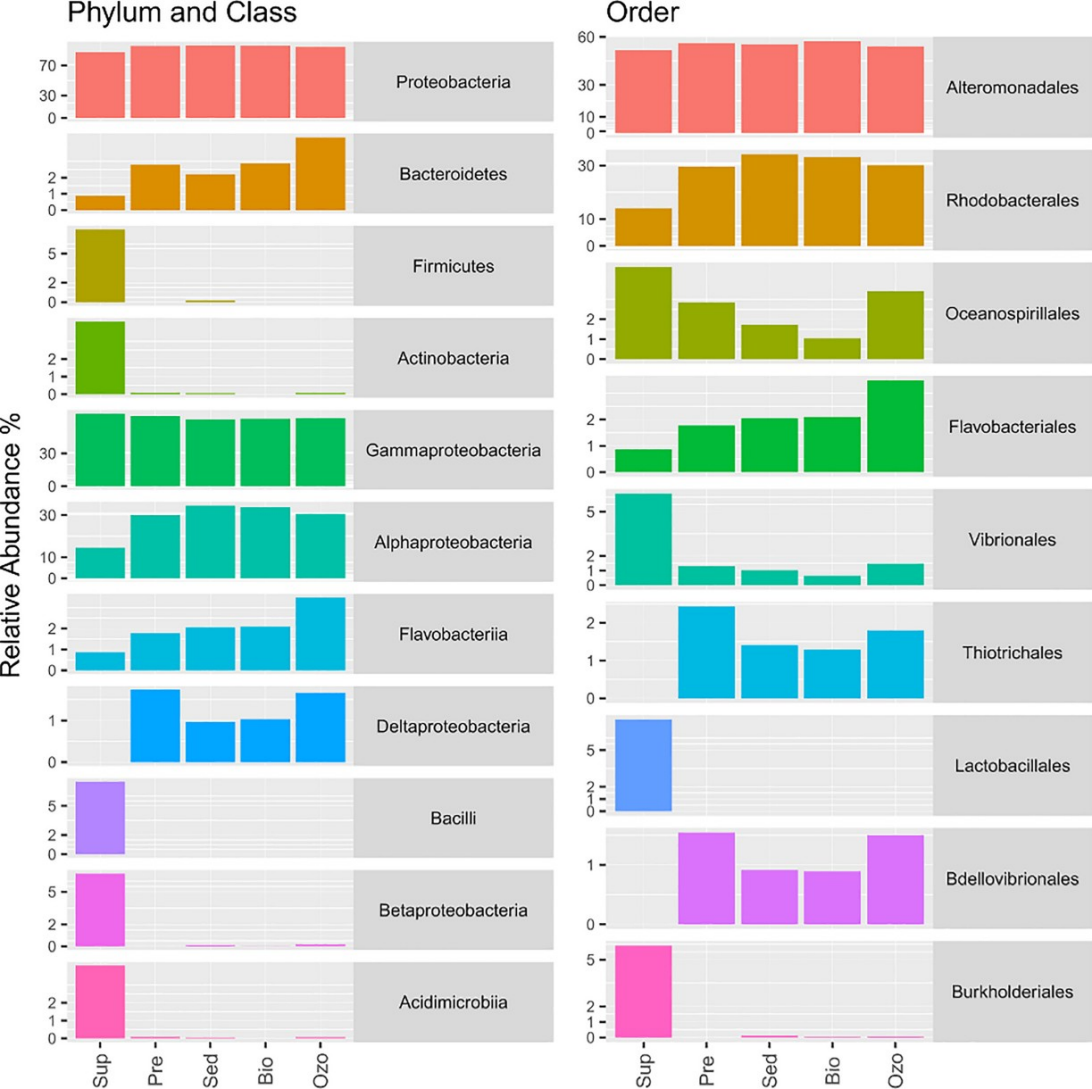
Relative abundance of the most dominant bacterial groups (4 phyla, 7 classes, 9 orders) in each sampling compartment.

**Fig 5:**
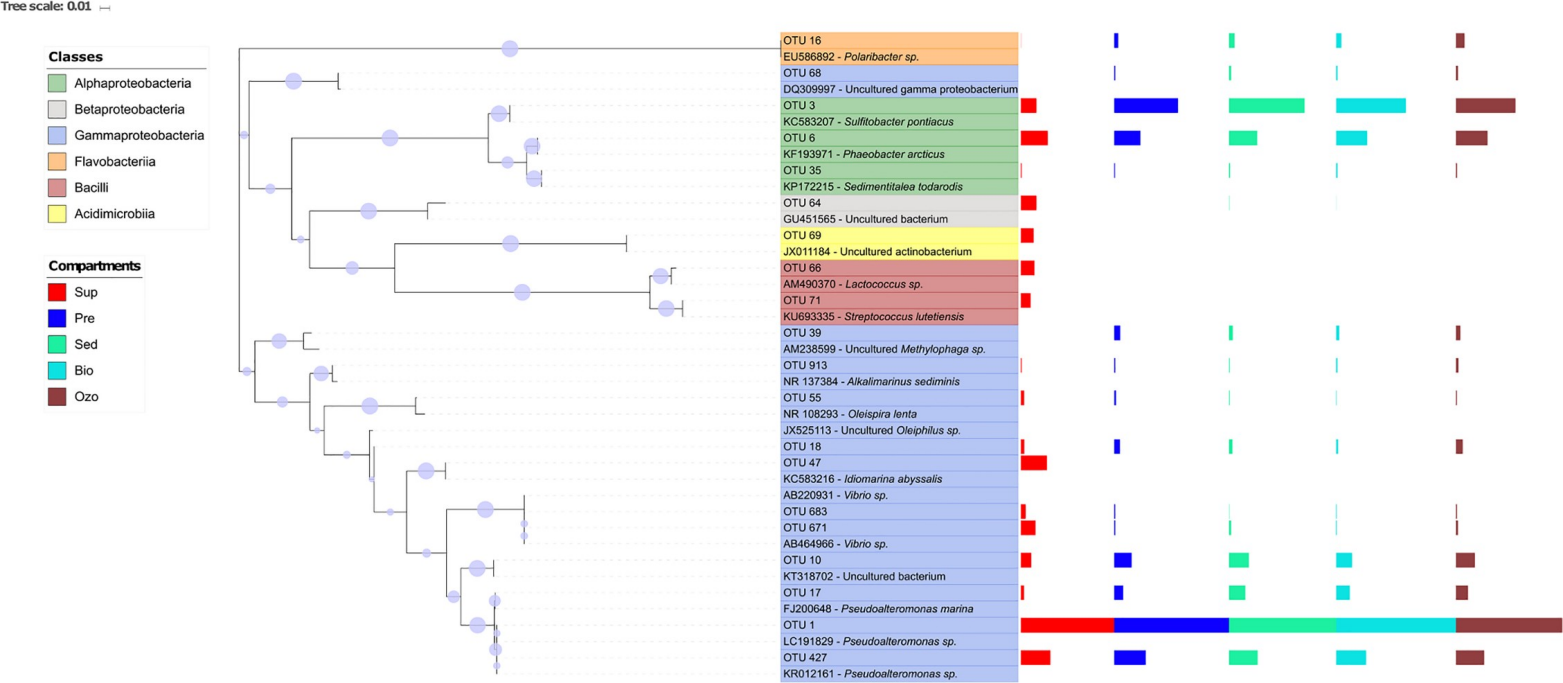
Phylogenetic tree of the most abundant OTUs (≥ 50 sequences) and their closest relatives in the GenBank (accession numbers are provided). The bar plots indicate the abundance of each OTU; with each compartment aligned with the maximum value of the previous compartment. Node confidence (1000 bootstrap replicates) higher than 50% is shown with symbol size (○) scaled to reflect support levels. Sup—water supply, Ozo—ozonation tank, Pre—pre-production tank, Sed—sedimentation tank and Bio—biofilter tank.

In this study, we used the RDP classification to obtain taxonomic information about the most abundant OTUs (≥50 sequence reads—Fig 4) and phylogenetic analyses to identify ecotypes related to these OTUs in different RAS compartments (Fig 5, S1 Table). This approach allowed us to better understand the composition and putative ecological role of the dominant bacterial populations in the RAS bacterioplankton.

The overall taxonomic analyses showed that Proteobacteria was the most abundant bacterial phylum in all RAS compartments (average relative abundance 94.60±4.10%), followed by Bacteroidetes (average relative abundance 2.65±1.30%*)* (Fig 4). The phyla Firmicutes and Actinobacteria were more abundant in the water supply (7.50% and 4.13%, respectively) than in the hatchery RAS (0.04% and 0.05%, respectively). The most abundant orders detected in this study were Alteromonadales (54.98±2.16%), Rhodobacterales (28.22±8.17%), Oceanospirillales (2.73±1.41%), Vibrionales (2.14±2.32%), Flavobacteriales (2.05±0.94%), Lactobacillales (1.50±3.35%), Thiotrichales (1.38±0.89%), Burkholderiales (1.22±2.62%) and Bdellovibrionales (0.97±0.62%) (Fig 4). Only 1.79±0.28% OTUs remained unclassified at the order level. Interestingly, the most abundant orders detected in the hatchery (Alteromonadales, Rhodobacterales, Oceanospirillales, Vibrionales, Flavobacteriales and Thiotrichales) were also the most abundant groups in our previous study on sole grow-out RAS [12]. In both studies, Alteromonadales was by far the most abundant order in the bacterioplankton. This order comprises copiotroph bacteria with wide distribution in marine environments [33]. In line with the higher concentration of nitrate in the hatchery tanks, previous studies suggest that Alteromonadales have a relevant environmental role in the uptake of nitrate in marine environments [34]. Probably, members of this order were enriched in the RAS due to high nutrient inputs from fish feed and fish exudates during intensive fish production. Most of the OTUs assigned within the Alteromonadales belonged to the *Pseudoalteromonas* genus (47.39±4.44%). Members of this genus include a large and cosmopolitan group of marine bacteria that are usually found in association with marine eukaryotes [35]. The genus *Pseudoalteromonas* contains numerous marine species that synthesize biologically active molecules and produce anti-bacterial products [36]. They have also been shown to exhibit specific activity against *Vibrio* spp. in aquaculture systems [37–40] and previous studies propose that members of this genus may comprise valuable biocontrol strains for application in aquaculture [36, 41].

In contrast to our previous study [12], a much higher abundance of Rhodobacterales was observed in the hatchery RAS. Members of this order are well known for their metabolic versatility (e.g. photosynthesis, CO_2_ and nitrogen fixation and sulfur oxidation) which can significantly contribute for nutrient cycling and improve water quality [42, 43]. Previous studies suggest that the Roseobacter clade (Rhodobacterales) may play an important role against the development of fish pathogens in aquaculture systems [44, 45]. For example, D'Alvise *et al*. (2010) [46] showed that a Vibrio-antagonistic Roseobacter (producer of tropodithietic acid, TDA), was able to suppress the development of the fish pathogen *Vibrio anguillarum* in model systems simulating a fish larval aquaculture environment. The most abundant OTUs assigned to Rhodobacterales (OTUs 3, 6 and 35) were present in all RAS compartments including water supply (Fig 5). However, OTU 3, the second most abundant OTU in the aquaculture system, was more abundant inside the hatchery tanks (21.40±2.40%) than in the water supply (4.95%). This OTU was similar to an organism previously identified as *Sulfitobacter pontiacus* (sequence similarity 100%, S1 Table). This species is specialized in sulfite oxidation and was detected for the first time in the black sea [47]. Several studies have reported on the occurrence of Sulfidobacteria in aquacultures, or nearby water, highlighting the potential importance of members of this genus in the sulfur cycling within these systems [48, 49]. Interestingly, Sharifah and Eguchi (2012) [50] showed that, in the presence of the phytoplankton *Nannochloropsis oculata*, *Sulfitobacter* sp. showed inhibitory activity towards *Vibrio anguillarum*. OTUs 6 and 35 showed close phylogenetic relationship to *Phaeobacter arcticus* and *Sedimentitalea todarodis* (members of the Roseobacter clade) and were abundant in the water supply (8.60% and 0.24%, respectively) and in the hatchery tanks (average relative abundance 9.34±0.78% and 0.36±0.06%, respectively) (Fig 5 and S1 Table, sequence similarities 100%). These species are described as psychotrophic bacteria previously isolated from Artic marine sediment (*P*. *arcticus*) and from the intestinal tract of a squid (*S*. *todarodis*) [51, 52]. Curiously, a previous study also detected these bacteria as abundant members of a marine RAS [53], however, there is no previous information about their putative role in aquaculture systems.

The variation in the relative abundance of the phylum Firmicutes was mainly related to OTUs 66 and 71 that were similar to organisms retrieved from a fish farm and from fish gut (sequences similarity = 99% and 100%, respectively) (Fig 5 and S1 Table). OTU 66 was assigned to the genus *Lactococcus* and OTU 71 to the genus *Streptococcus*. Members of these genera belong to the lactic acid bacteria group and are often found in fish guts [54]. In this study, they were only detected in the water supply (OTU 66–4.37% and OTU 71–3.12%), which could indicate limited ability to colonize the water of hatchery RAS. The Actinobacteria phylum was dominated by OTU 69 (close related to uncultured actinobacterium from seawater) and was only detected in the water supply (Fig 5 and S1 Table). Members of the Oceanospirillales order were present in all compartments (including water supply) and were mainly represented by OTUs 18 (1.35±0.6%) and 55 (0.47±0.38%) (Fig 5, S1 Table). Members of the Oceanospirillales are often described as halotolerant and halophilic, aerobic, microaerophilic or facultative chemoorganotrophs and are widespread in marine environments [55]. OTU 55 was similar to an organism previous identified as *Oleispira lenta* (sequence similarity = 99%) (Fig 5, S1 Table). Members of this species have been described as mesophilic hydrocarbon degraders [56]. A recent study reported on the dominance of an OTU assigned to the genus *Oleispira* associated with salmon skin [57], which could indicate their ability to colonize fish skin.

Flavobacteriales were more abundant in the hatchery RAS (2.32±0.77%) than in the water supply (0.89%). Flavobacteriales was mainly represented by OTU 16 (average relative abundance of 1.87±0.63% inside the RAS), which was assigned to the Flavobacteriaceae family and was similar to an organism previously identified as *Polaribacter* sp. (Fig 5 and S1 Table, sequence similarity 100%) obtained from aquaculture water. This OTU was also present in the water supply but showed much higher abundance in the RAS tanks. Members of this genus have been found in RAS compartments in different geographic locations [12, 58, 59]. Rud *et al*. (2016) [59], specifically, found a higher abundance of *Polaribacter* sp. in tank biofilms when compared to water in a RAS system. Members of the Flavobacteriales are known for their ability to form biofilms on surfaces in marine environments [60, 61]. Such ability may improve their capacity to colonize the RAS environment.

The orders Thiotrichales and Bdellovibrionales were only detected inside the hatchery RAS (average relative abundance of 1.72±0.51% and 1.21±0.36%, respectively) (Fig 4). The order Thiotrichales was mainly represented by OTU 39 (average relative abundance of 1.40±0.40% inside of the RAS), which was assigned to the Piscirickettsiaceae family (S1 Table). This OTU was only 96% similar to its closest relative in the GenBank database, an uncultured *Methylophaga sp*. (Fig 5). Members of this genus have been described as aerobic methylotrophs involved in denitrification in marine environments, seawater aquariums and aquacultures [48, 62]. The high abundance of Bdellovibrionales (Fig 4) is also noteworthy, since members of this order prey exclusively on other bacteria including potential fish pathogens [63, 64]. Bdellovibrionales and similar organisms (BALOs) isolated from fish ponds have been shown to reduce disease incidence caused by the fish pathogens *Aeromonas hydrophila* and *Vibrio alginolyticus* ([65] and references therein). The orders Vibrionales and Burkholderiales were both moderately abundant in the water supply (6.25% and 5.91%, respectively), however, their abundance was reduced in the hatchery compartments (1.11±0.36% and 0.05±0.04%, respectively). Overall, our results showed that, with exception of OTUs 39 and 68, all dominant OTUs detected in the hatchery tanks were originally present in the water supply before entering the RAS.

In order to evaluate the composition of potential fish pathogens in the hatchery RAS we also specifically searched for OTUs related to bacterial genera which often comprise known fish pathogens (S2 Table). OTUs 49 and 198 (0.17±0.14% and 0.02±0.02%, respectively) were assigned to *Vibrio ichthyoenteri* (S2 Table, sequence similarities of 100%). This species was previously reported to be a pathogen of flounder (*Paralichthys olivaceus*) [66]. Likewise, OTU 70 (relative abundance 0.06±0.03%) was similar to a microorganism identified as *Vibrio anguillarum* (S2 Table), a pathogen that causes vibriosis in approximately 50 species of fish [67]. However, it should be noted that despite the 16S rRNA gene can be used for classification of *Vibrio* at genus level, this gene may not have enough resolution for *Vibrio* at the species level [12, 68] and must be carefully considered when used to interpret the diversity of *Vibrio* communities. Interestingly, despite the relatively high abundance of members of the Vibrionales order in the water supply, only a few members of this genus found favorable conditions inside the hatchery RAS (Fig 4). OTUs 59 and 290 were assigned to *Serratia marcescens* and *Francisella philomiragia*, respectively, two known fish pathogens (S2 Table, sequences similarities of 100%). These OTUs occurred in low abundance inside the RAS and only *Francisella philomiragia* was detected in the fish compartment (Pre). This species is an opportunistic waterborne pathogen able to cause disease in a range of animals, including finfish species [69, 70]. However, in line with our previous study [12] and despite the presence of potential pathogens, no disease outbreak occurred in the hatchery RAS during this study.

## Conclusion

Exploring the potential of naturally occurring microorganisms as biocontrol agents in aquacultures is not a new concept [46, 53, 71, 72]. The development of microbial management or modulation approaches should be based on a fundamental knowledge about the aquaculture microbiome. This study provides baseline information about the bacterioplankton community composition and diversity of a commercial hatchery RAS for the production of juvenile Senegalese sole. Our results showed that despite the differences in relative abundance, the most abundant orders detected in the hatchery RAS (Alteromonadales, Rhodobacterales, Oceanospirillales, Vibrionales and Flavobacteriales) were also the most abundant detected in the sole grow-out RAS characterized in our previous study [12]. Curiously, in contrast to our findings for grow-out RAS, our results indicated that the bacterial assemblage of the water supply played an important role for the colonization of bacterial populations (e.g. *Pseudoalteromonas sp*., members of the Roseobacter clade, *Phaeobacter arcticus* and *Sedimentitalea todarodis* and Sulfidobacteria) in the hatchery RAS. Most remarkable, here water supply seems to contribute for a strong colonization of *Pseudoalteromonas sp*. in the tanks, which in turn may play a role in suppressing the development of potential fish pathogens in the aquaculture system [36–40]. Our findings suggest that the bacterial community of the water supply may influence the bacterioplankton community structure of sole hatchery RAS. However, taking in consideration the results obtained for sole grow out RAS [12], the contribution of water supply to shape RAS bacterioplankton communities may vary between different RAS. Further studies are needed to investigate the effect of reared fish species and aquaculture practices for identification of the key drivers of RAS bacterioplankton communities.

## Supporting information

S1 Fig DGGE profiles of 16S rRNA gene amplified from total community DNA extracted from three replicates of water supply (Sup), ozonation tank (Ozo), biofilter tank (Bio), pre-production (hatchery) tank (Pre) and sedimentation tank (Sed). (TIFF)

S1 Table List of most abundant bacterial OTUs across the dataset (≥50 sequences) and their relative abundance in water supply (Sup), sole pre-production tank (Pre), sedimentation tank (Sed), biofilter tank (Bio) and ozone tank (Ozo). The table includes the taxonomic assignment, the closest related organisms using BLAST, their accession numbers, the sequence similarity of the closest matches with our representative OTU sequences (SEQ) and the source of these organisms.
(PDF)

S2 Table List of OTUs related to bacterial genera which often comprise potential fish pathogens and their relative abundance in water supply (Sup), sole pre-production tank (Pre), sedimentation tank (Sed), biofilter tank (Bio) and ozone tank (Ozo). The table includes the taxonomic assignment, the closest related organisms using BLAST, their accession numbers, the sequence similarity of the closest matches with our representative OTU sequences (SEQ) and the source of these organisms.
(PDF)
