# Supplementary figures and images for: Characterization of bacterioplankton communities from a hatchery recirculating aquaculture system (RAS) for juvenile sole (Solea senegalensis) production
Source: PLoS One. 2019 Jan 25;14(1):e0211209. doi: 10.1371/journal.pone.0211209 (PMC6347143; doi:10.1371/journal.pone.0211209)

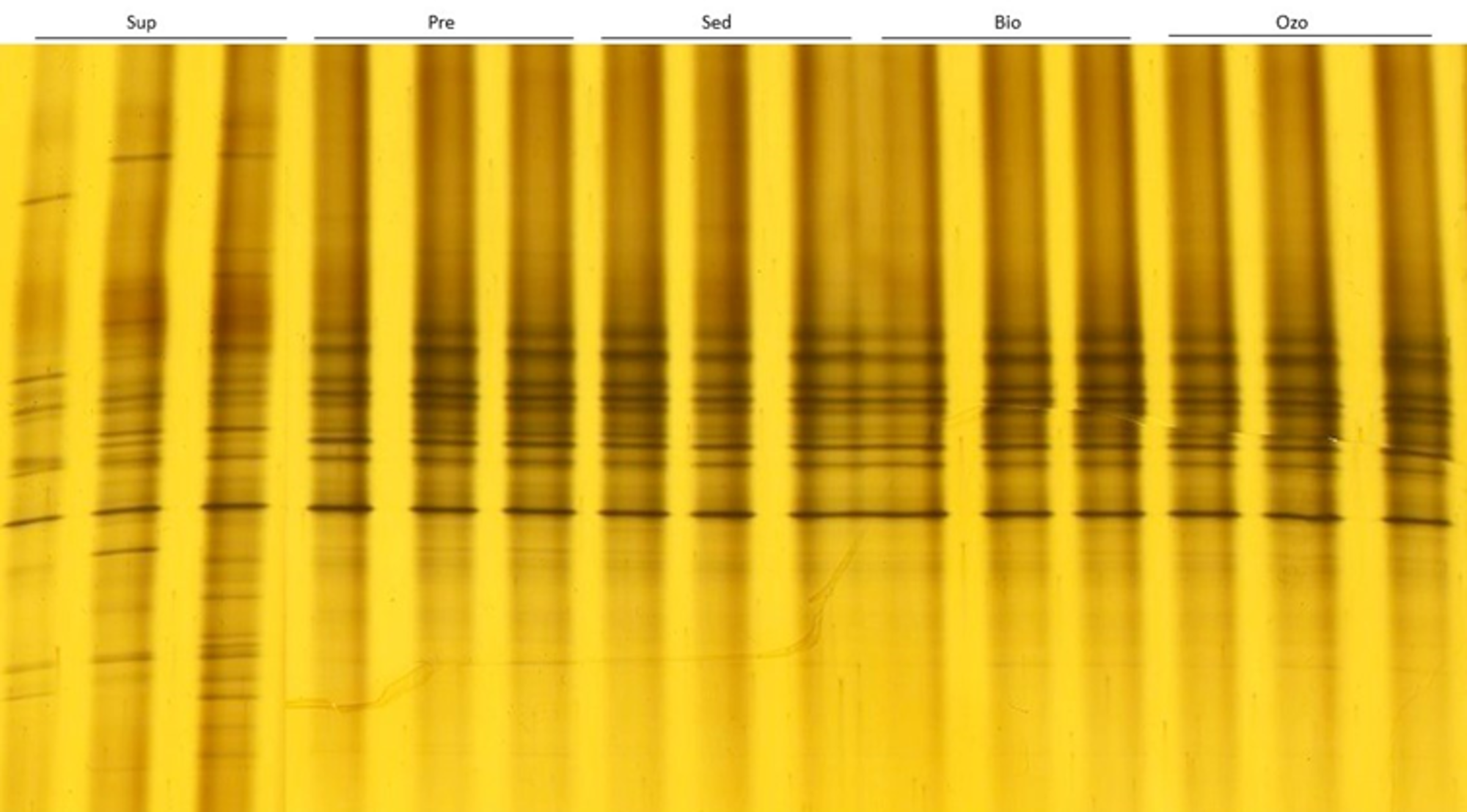

Supplement: S1 Fig — (TIFF) [file pone.0211209.s001.tiff]
