# Supplementary material for: Characterization of bacterioplankton communities from a hatchery recirculating aquaculture system (RAS) for juvenile sole (Solea senegalensis) production
Source: PLoS One. 2019 Jan 25;14(1):e0211209. doi: 10.1371/journal.pone.0211209 (PMC6347143; doi:10.1371/journal.pone.0211209)
Supplement: S1 Table — The table includes the taxonomic assignment, the closest related organisms using BLAST, their accession numbers, the sequence similarity of the closest matches with our representative OTU sequences (SEQ) and the source of these organisms. (PDF) [file pone.0211209.s002.pdf]

Table S1 List of most abundant bacterial OTUs across the dataset (≥50 sequences) and their relative abundance in water supply (Sup), sole pre-production tank (Pre[illegible] sedimentation tank (Sed), biofilter tank (Bio) and ozone tank (Ozo). The table includes the taxonomic assignment, the closest related organisms using BLAST, their accession numbers, the sequence similarity of the closest matches with our representative OTU sequences (SEQ) and the source of these organisms.

OTU	SUP	PRE	SED	BIO	OZO	PHYLUM	CLASS	ORDER	FAMILY	GENUS	ACCESSION NUMBER	SEQ	SOURCE
**1**	29.70	36.55	34.18	38.08	33.90	Proteobacteria	Gammaproteobacteria	Alteromonadales	Pseudoalteromonadaceae	Pseudoalteromonas	LC191829	100	seawater, Japan
**3**	4.95	20.34	24.11	22.18	18.96	Proteobacteria	Alphaproteobacteria	Rhodobacterales	Rhodobacteraceae	Sulfitobacter	KC583207	100	seawater from Rio Grande Rise Region, South Atlantic
**6**	8.60	8.41	8.99	9.87	10.08	Proteobacteria	Alphaproteobacteria	Rhodobacterales	Rhodobacteraceae	Phaeobacter	KF193971	100	gastrointestinal tract of farmed olive flounder (*Paralichthys olivaceus*), South Korea
**10**	3.32	5.56	6.35	5.05	6.02	Proteobacteria	Gammaproteobacteria	Alteromonadales	Unclassified	Unclassified	KT318702	100	ocean water from northeastern Gulf of Mexico (after exposure to oil and dispersant)
**16**	0.05	1.31	1.78	1.62	2.77	Bacteroidetes	Flavobacteriia	Flavobacteriales	Flavobacteriaceae	Polaribacter	EU586892	100	RAS seawater, Portugal
**17**	1.01	2.86	5.22	4.30	3.87	Proteobacteria	Gammaproteobacteria	Alteromonadales	Pseudoalteromonadaceae	Pseudoalteromonas	FJ200648	100	seawater from Turkey: eastern Aegean Sea
**18**	1.11	1.81	1.13	0.56	2.19	Proteobacteria	Gammaproteobacteria	Oceanospirillales	Oleiphilaceae	uncultured Oleiphilus	JX525113	99	surface water from the Southern ocean (iron fertilization experiment), India
**35**	0.24	0.27	0.38	0.36	0.42	Proteobacteria	Alphaproteobacteria	Rhodobacterales	Rhodobacteraceae	Sedimentitalea	KP172215	100	Japanese flying squid (*Todarodes pacificus),* South [illegible]
**39**	0.00	1.93	1.24	0.98	1.45	Proteobacteria	Gammaproteobacteria	Thiotrichales	Piscirickettsiaceae	uncultured Methylophaga	AM238599	96	sea water enriched with dimethylsulfide, Atlant[illegible] Ocean: Pensacola Pier
**47**	8.31	0.00	0.00	0.00	0.00	Proteobacteria	Gammaproteobacteria	Alteromonadales	Idiomarinaceae	Idiomarina	KC583216	100	Oceanic water from Rio Grande Rise Region, [illegible] Ocean, Brazil
**55**	1.06	0.62	0.22	0.11	0.35	Proteobacteria	Gammaproteobacteria	Oceanospirillales	Oceanospirillaceae	Oleispira	NR108293	99	Coastal seawater from Yellow Sea
**64**	4.95	0.00	0.11	0.03	0.00	Proteobacteria	Betaproteobacteria	Burkholderiales	Unclassified	Unclassified	GU451565	97	macroalgal surface
**66**	4.37	0.00	0.00	0.00	0.00	Firmicutes	Bacilli	Lactobacillales	Streptococcaceae	Lactococcus	AM490370	99	fish and the fish farm environment
**68**	0.00	0.42	0.70	0.36	0.71	Proteobacteria	Gammaproteobacteria	Unclassified	Unclassified	Unclassified	DQ309997	99	associated with the red seaweed, *Delisea pulchra. Australia*
**69**	4.08	0.00	0.00	0.00	0.00	Actinobacteria	Acidimicrobiia	Acidimicrobiales	C111	Unclassified	JX011184	100	Marine sample, China
**71**	3.12	0.00	0.00	0.00	0.00	Firmicutes	Bacilli	Lactobacillales	Streptococcaceae	Streptococcus	KU693335	100	Lactic acid bacteria from fish gut, Thailand
**427**	9.42	10.07	9.15	9.48	8.99	Proteobacteria	Gammaproteobacteria	Alteromonadales	Pseudoalteromonadaceae	Pseudoalteromonas	KR012161	100	Deep-sea sediment from the Pacific Ocean
**671**	4.66	0.42	0.70	0.17	0.71	Proteobacteria	Gammaproteobacteria	Vibrionales	Vibrionaceae	Aliivibrio	AB464966	100	Senegal sole (*Solea senegalensis*) intestine, Spain: [illegible]
**683**	1.54	0.35	0.11	0.14	0.39	Proteobacteria	Gammaproteobacteria	Vibrionales	Vibrionaceae	Unclassified	AB220931	100	Intestine of japanese flounder (Paralichthys oliv[illegible] Japan
**913**	0.24	0.39	0.22	0.33	0.85	Proteobacteria	Gammaproteobacteria	Oceanospirillales	Oleiphilaceae	Alkalimarinus	NR_137384	99	Marine sediment, China: Weihai coast
