# Supplementary material for: Characterization of bacterioplankton communities from a hatchery recirculating aquaculture system (RAS) for juvenile sole (Solea senegalensis) production
Source: PLoS One. 2019 Jan 25;14(1):e0211209. doi: 10.1371/journal.pone.0211209 (PMC6347143; doi:10.1371/journal.pone.0211209)
Supplement: S2 Table — The table includes the taxonomic assignment, the closest related organisms using BLAST, their accession numbers, the sequence similarity of the closest matches with our representative OTU sequences (SEQ) and the source of these organisms. (PDF) [file pone.0211209.s003.pdf]

Table S2 List of OTUs related to bacterial genera which are often comprising potential fish pathogens and their relative abundance in water supply (Sup), sole pre-production tank (Pre), sedimentation tank (Sed), biofilter tank (Bio) and ozone tank (Ozo). The table includes the taxonomic assignment, the closest related organisms using BLAST, their accession numbers, the sequence similarity of the closest matches with our representative OTU sequences (SEQ) and the source of these organisms.

OTU	SUP	PRE	SED	BIO	OZO	CLASS	ORDER	FAMILY	GENBANK	Accession Number	SEQ	SOURCE
49	0.00	0.39	0.11	0.20	0.16	Gamma	Vibrionales	Vibrionaceae	*Vibrio ichthyoenteri*	AM181658	100	digestive tract of *Paralichthys olivaceus*
59	0.00	0.00	0.00	0.03	0.00	Gamma	Enterobacteriales	Enterobacteriaceae	*Serratia marcescens*	KT215434	100	freshwater
70	0.00	0.08	0.05	0.03	0.09	Gamma	Vibrionales	Vibrionaceae	*Vibrio anguillarum*	KR270138	100	gut of *Apostichopus japonicus*
198	0.00	0.04	0.00	0.03	0.05	Gamma	Vibrionales	Vibrionaceae	*Vibrio ichthyoenteri*	HG931133	100	cultured Sparus aurata
208	0.00	0.00	0.05	0.00	0.00	Gamma	Vibrionales	Vibrionaceae	*Vibrio sp.*	EU253597	100	Mediterranean Sea surface water
290	0.00	0.04	0.00	0.00	0.00	Gamma	Legionellales	Francisellaceae	*Francisella philomiragia*	EF364047	100	farmed Atlantic cod
544	0.00	0.00	0.00	0.03	0.02	Gamma	Vibrionales	Vibrionaceae	*Vibrio splendidus*	KF009796	100	Portugal seawater
671	4.66	0.42	0.70	0.17	0.71	Gamma	Vibrionales	Vibrionaceae	*Vibrio sp.*	AB464966	100	sole intestine
683	1.54	0.35	0.11	0.14	0.39	Gamma	Vibrionales	Vibrionaceae	*Vibrio sp.*	AB220931	100	coastal seawater
1140	0.00	0.00	0.00	0.03	0.00	Flavo	Flavobacteriales	Flavobacteriaceae	*Flavobacterium sp.*	KT284905	97	soil of rhizosphere seepweed
